# Modern drug self-medication and associated factors among pregnant women at Settat city, Morocco

**DOI:** 10.3389/fphar.2022.812060

**Published:** 2022-08-16

**Authors:** Samia Chergaoui, Omaima Changuiti, Abdelghafour Marfak, Elmadani Saad, Abderraouf Hilali, Ibtissam Youlyouz Marfak

**Affiliations:** ^1^ Laboratory of Health Sciences and Technologies, Higher Institute of Health Sciences, Hassan First University of Settat, Settat, Morocco; ^2^ National School of Public Health, Rabat, Morocco

**Keywords:** pregnancy, self-medication, modern drug, OTC medication, maternal and child health

## Abstract

**Purpose:** The consumption of drugs during pregnancy without medical advice constitutes a risk for the mother and the fetus. It is a public health problem. This study aimed to assess self-medication practices among pregnant women, the most used medicines, and factors associated with this practice.

**Methods:** A cross-sectional study was conducted using a structured questionnaire on pregnant women who were attending Settat health centers. A simple random sampling technique was used to select the study participants. Descriptive and inferential statistics were computed using the SPSS version 19.

**Results:** Among 364 pregnant women, 118 (32%) practiced self-medication in modern medicine. Paracetamol was the most used medication, and nausea and vomiting were the most frequent symptoms reported by self-medicated pregnant women. Multivariate logistic regression analysis showed that women over 30 years old were four-fold more likely to practice self-medication than the other groups [AOR: 4.19; 95% CI (1.80–9.77)]. Similarly, unemployed women [AOR: 3.93; 95% CI (0.80–19.23)], those in third trimester [AOR: 2.63; 95% CI (1.29–5.36)], multiparous [AOR: 6.03; 95% CI (3.12–11.65)], without chronic illness [AOR: 2.84; 95% CI (1.26–6.41)], without therapeutic treatment [AOR: 10.1; 95% CI (2.81–37.03)] and who have attended ANC at least once, were more likely to practice self-medication than the other groups.

**Conclusion:** The prevalence of modern drug self-medication among pregnant women in Morocco is classified as lower. Health professionals can exert positive pressure through education and information provided during ANC about OTC medications to significantly reduce the rate of self-medication.

## Introduction

Self-medication is defined as the use of medication to treat self-diagnosed disorders or symptoms or the intermittent or continuous use of prescribed medication for chronic or recurring illnesses or symptoms (World Health Organization, 2000). This practice is widespread in the world, and its overall estimated prevalence was 32% (Mohseni et al., 2018). Several studies have shown that self-medication is very common in countries with limited resources, such as African countries. There, people use both over-the-counter and prescription drugs without supervision (Afolabi, 2008; Aziz et al., 2018; Marwa et al., 2018). What makes the difference between countries is mainly differences in health policy, access to care, and regulation of drug distribution (Branthwaite and Pechère, 1996).

Many studies have shown an association between self-medication and socio-demographic, cultural, and economic characteristics (Subashini and Udayanga, 2020; Zeid and al., 2020). Morocco is one of the developing countries where the prevalence of self-medication is 69%, of which 64.3% is represented by women in general (Oirdi et al., 2015). Very few studies have evaluated self-medication in Morocco, but none have studied self-medication among pregnant women.

Pregnant women are one of the vulnerable population groups practicing the two forms of self-medication (90%), either over-the-counter (OTC) or prescribed medications. Furthermore, studies have shown that up to 10% of birth defects (low birth weight, premature birth, feeding problems, respiratory problems, malformations, and developmental fetal toxicity) are directly linked to maternal drug exposure (Stanley et al., 2019).

In fact, as far as the drugs are concerned, some of them have been documented as safe for use during pregnancy. However, research cannot describe all the drugs used by pregnant women in self-medication for ethical concerns. Thus, in 2015, the United State Food and Drug Administration (US-FDA) implemented the Pregnancy Lactation Labeling Rule (PLLR), which requires reformulation of the content and format of drug packaging. This reformulation included a descriptive summary of the risks associated with the use of medications during pregnancy and lactation. The US-FDA authorizes a drug product based on clinical trial data using controlled groups, random assignment of participants to groups, techniques to minimize bias, and defined criteria to measure outcomes. For pregnant women, the safety of a drug means that the drug is not known to be embryonical, teratogenic, or fetotoxic, and indicates that the drug is safe for the woman herself (Food and Drug Administration, 2014; United States Food and Drug Administration, 2015).

To overcome all kinds of disorders caused by self-medication during pregnancy, health professionals involved in prenatal surveillance (general practitioners, gynecologists–obstetricians, and midwives) must have a role in preventing these risks through monitoring and raising awareness. Thus, the information provided by health professionals is a decisive lever in the prevention of risks due to self-medication (Haute Autorité de Santé, 2007).

## Material and methods

### General context of the study

The reduction of maternal and neonatal morbidity and mortality is among the goals of the international community. In Morocco, the maternal mortality ratio has decreased from 227 deaths per 100,000 live births in 2004 to 72.6 per 100,000 births in 2018. It is twice as high in rural areas (111 per 100,000 births) than in urban areas (45 per 100,000). This progress is explained, in part, by improved prenatal care. The latter makes it possible to assess and anticipate possible risks to future pregnancy, such as exposure to occupational and lifestyle risks, including nutrition and self-medication (ENPSF, 2018).

### Study design and setting

A facility-based cross-sectional study was conducted to assess self-medication among pregnant women who were attending ANC at the seven Settat's health centers between January and July 2019. Settat, a Moroccan city located in the Casablanca-Settat region, is the capital of the province of Settat. In 2020, the population of Settat city reached 159,002 inhabitants. The city’s health infrastructure comprises a provincial hospital (Hassan II Hospital) and seven primary health centers (Haut Commissariat au Plan (HCP), 2018). These centers are state facilities offering free services to the city’s population. They provide primary health care and contain a maternal and child health service, where prenatal consultations are performed.

### Population and inclusion and exclusion criteria

All pregnant women who had ANC follow-up in one of Settat’s health centers were the source population. Pregnant women of any gestational age who came during the study period were included in this study. Pregnant women who were unable to hear or communicate and those who had given birth and were unwilling to provide consent were excluded from the study.

### Sampling procedure

Before starting the study, we contacted all seven health centers. Each center informed us of the number of pregnant women coming for ANC. A list of contact information for pregnant women attending each health center was provided. The seven lists were compiled in an Excel file. In total, there were 3,900 women.

The sample size was estimated using the following single proportion formula:

$$n=\frac{\mathrm{N*}Z_{\alpha }^{2}p\left(1-p\right)}{\left(N-1\right)\varepsilon^{2}+Z_{\alpha }^{2}p\left(1-p\right)}.$$

Since no study has reported on modern drug self-medication among Moroccan pregnant women, we proposed the prevalence of self-medication, *p* = 50%. Considering a margin error of 5% and a confidence level of 95%, the minimum number of participants to be included in this study was *n* = 350. Expecting a 10% non-response rate, we interviewed a total of 384 pregnant women. The final sample size was set at 364 participants. The sampling was carried out by the simple random method using the function RANDBETWEEN (1; 3,900) to select the 384 women to be interviewed. We called these women by phone to schedule appointments with them during their subsequent visits.

### Data collection

Data were collected using a structured questionnaire developed in French and translated into the Moroccan local dialect (Darija). The questionnaire was administered face-to-face by a single interviewer. A pre-test was carried out on 5% of the sample at the health center Al Kheir of Settat. The reliability of the questionnaire was also checked using Cronbach’s alpha test, and a value of 0.80 was obtained. The questionnaire included socio-demographic characteristics, obstetric factors, and self-medication practice. Regarding self-medication, women in the first trimester of their pregnancy answered the questions during this period, those in the second trimester answered the questions for the second and first trimesters, and those in the third trimester answered the questions concerning the three trimesters. The participants collaborated in our study on a voluntary basis. They provided their written consent form after they were assured of the anonymity of the questionnaires, the confidentiality of their data, and their right to withdraw at any time during the interviews. Data were collected for each pregnant woman, noted on copies of the questionnaire, and then transcribed in an Excel file which served as the basis for statistical analysis.

### Statistical analysis

Data analysis was performed using Software Statistical Package for the Social Science (SPSS) version 19. Qualitative variables were presented as frequency and percentage. The dependent variable was the modern drug self-medication, and the independent variables were socio-demographic characteristics of pregnant women. The comparison between categorical variables and self-medication practice was analyzed using Fisher’s exact test and a Chi-square test. Multicollinearity was checked out to test correlation among associated variables using the variance inflation factor (VIF). Bivariate logistic regression analysis was used for examining the association between self-medication and socio-demographic characteristics. Variables with a *p*-value less than 0.05 were re-analyzed using a multivariable binary logistic regression model to identify the factors independently associated with modern drug self-medication practice during pregnancy. The strength of associations between each exposure variable and the dependent variable was measured by odds ratios (OR) with 95% confidence intervals (CIs) and statistical significance was set at *p* < 0.05.

## Results

### Socio-demographic characteristics

A total of 364 pregnant women met the inclusion criteria. Among them, 355 (97.5%) were married, and 180 (49.5%) were in the age range 20–30 years. Moreover, 344 (94.5%) participants were unemployed, 109 (29.9%) were illiterate, and 107 (29.4%) were on the primary level. Furthermore, 295 (81%) of the pregnant women had no chronic illness and 312 (85.7%) were not on any treatment (Table 1).

**TABLE 1:** Socio-demographic and obstetrical characteristics and self-medication during pregnancy of 364 women, 2019.

Characteristic		Self-medication			*p*-value
		Total, *N* = 364 (%)	Yes, *N* = 118 (%)	No, *N* = 246 (%)	
Age	18–20	59	11 (9.3)	48 (19.5)	**<0.0001**
	20–30	180	44 (37.3)	136 (55.3)	
	30–45	125	63 (53.4)	62 (25.2)	
Residence	Urban	364	118	246	—
	Rural	0	0	0	
Education	College/University	42	16 (13.6)	26 (11.1)	0.403
	Secondary school	93	36 (30.8)	57 (24.4)	
	Primary school	107	34 (29.1)	73 (31.2)	
	Illiterate	109	31 (26.5)	78 (33.3)	
Occupation	Employed	20	2 (1.7)	18 (7.3)	**0.028**
	Unemployed	344	116 (98.3)	228 (92.7)	
Chronic illness	Yes	69	11 (9.3)	58 (23.6)	**0.001**
	No	295	107 (90.7)	188 (76.4)	
Therapeutic treatment	Yes	52	3 (2.5)	49 (19.9)	**<0.0001**
	No	312	115 (97.5)	197 (80.1)	
Parity	Primiparous	168	38 (32.2)	130 (52.8)	**<0.0001**
	Second parous	110	33 (28.0)	77 (31.3)	
	Multiparous	86	47 (39.8)	39 (15.9)	
Gestational age	First trimester	80	20 (16.9)	60 (24.3)	**0.01**
	Second trimester	157	44 (37.2)	113 (45.9)	
	Third trimester	127	54 (45.7)	73 (29.6)	
Previous ANC follow up	0 ANC	61	19 (16.1)	14 (5.69)	**0.004**
	1–3 ANC	97	32 (27.1)	87 (35.3)	
	4 ANC	206	67 (56.7)	145 (58.9)	

### Obstetric factors

Nearly half 157 (43.1%) of the participants were in the second trimester of their pregnancy, and 168 (46.2%) participants were primiparous. About 206 (56.5%) of participants had a full ANC follow-up in their previous pregnancy. The reasons for the lack of ANC follow-up were mainly financial and logistical resources 168 (46.1%) (Table 1).

### Modern drug self-medication

Globally, this study showed that modern drug self-medication is practiced during the pregnancy period by 118 (32%) women. Among them, 51 (43.2%) self-medicated during the first trimester, 24 (20.3%) only in the second trimester, and 43 (36.4%) in the third trimester.

**FIGURE 1:**
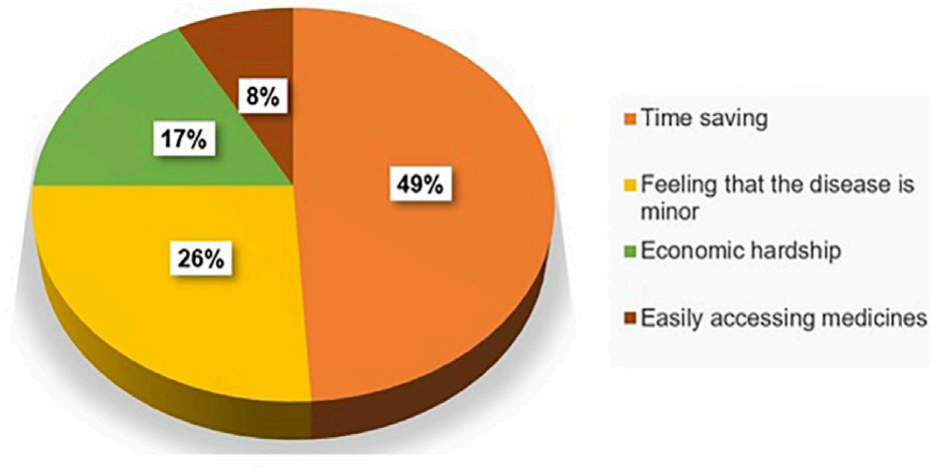
Self-medication reasons of study participants, 2019 (*n* = 118).

The most common reason for practicing self-medication was time-saving 67 (48.6%), followed by an assumption that the disease was minor 36 (26.1%) (Figure 1).

The common causes of self-medication were nausea and vomiting 83 (29.3%), headache 68 (24%), heartburn 67 (23.7%), and back and leg pain 65 (23%) (Table 2).

**TABLE 2:** Characteristics of modern drug self-medication among pregnant women at Settat, 2019 (*n* = 118).

Variables	Frequency (*N*)	Percentage (%)
Indication		
Nausea/Vomiting	83	70.3
Headache	68	57.6
Heartburn	67	56.7
Back and leg pain	65	55
Constipation	46	38.9
Cough	38	32.2
Diarrhea	17	14.4
Hemorrhoids	14	11.8
Common drugs used for self-medication		
Paracetamol	34	28.8
Ranitidine	25	21.1
Aspirin	23	19.4
Aluminum hydroxide/Magnesium hydroxide	23	19.4
Cough syrup/Pseudoephedrine hydrochloride	22	18.6
Sources of information		
Pharmacist	50	42.3
Themselves	40	33.8
Family or friends	28	23.7

As far as the drugs are concerned, 34 (28.8%) pregnant women self-administered paracetamol, 25 (21.1%) ranitidine, and 23 (19.4%) aspirin.

Among pregnant women who practice self-medication 118) with modern medicines during their current pregnancy, the most common sources of information were pharmacists 50 (42.3%) and by themselves 40 (33.8%) (Table 1).

Among the 364 participants, 227 (62.36%) were informed about the dangers and contraindications of self-medication during pregnancy.

Self-medication was associated with higher age (*p* < 0.0001), occupation (*p* = 0.028), chronic illness (*p* = 0.001), therapeutic treatment (*p* < 0.0001), parity (*p* < 0.0001), gestational age (*p* = 0.01), and previous ANC follow-up (*p* = 0.004).

Multivariate logistic regression analysis showed that women over 30 years old were four times more likely to practice self-medication than the other groups [AOR: 4, 19; 95% CI (1.80–9.77)]. Similarly, unemployed women [AOR: 3.93; 95% CI (0.80–19.23)], in third trimester [AOR: 2.63; 95% CI (1.29–5.36)], multiparous [AOR: 6.03; 95% CI (3.12–11.65)], without chronic illness [AOR: 2.84; 95% CI (1.26–6.41)], without therapeutic treatment [AOR: 10.1; 95% CI (2.81–37.03)] and who had attended ANC at least once were more likely to practice self-medication than the other groups (Table 3).

**TABLE 3:** Factors associated with modern drug self-medication of study participants, 2019 (*n* = 364).

Characteristic	Self-medication		COR [95% CI]	*p-value*	AOR [95% CI]	*p-value*
	Yes (%)	No (%)				
Age						
< 20	11 (9.3)	48 (19.5)	1.00	—	1.00	—
20–30	44 (37.3)	136 (55.3)	1.41 [0.67–2.95]	0.358	1.35 [0.59–3.12]	0.47
> 30	63 (53.4)	62 (25.2)	4.43 [2.11–9.32]	**<0.0001**	4.19 [1.80–9.77]	**0.001**
Occupation						
Employed	2 (1.7)	18 (7.3)	1.00	—	1.00	—
Unemployed	116 (98.3)	228 (92.7)	4.58 [1.05–20.07]	**0.028**	3.93 [0.80–19.23]	0.091
Chronic illness						
Yes	11 (9.3)	58 (23.6)	1.00	—	1.00	—
No	107 (90.7)	188 (76.4)	3.00 [1.51–5.96]	**0.001**	2.84 [1.26–6.41]	**0.011**
Therapeutic treatment						
Yes	3 (2.5)	49 (19.9)	1.00	—	1.00	—
No	115 (97.5)	197 (80.1)	9.53 [2.91–31.28]	**<0.0001**	10.1 [2.81–37.03]	**<0.0001**
Parity						
1	38 (32.2)	130 (52.8)	1.00	—	1.00	—
2	33 (28.0)	77 (31.3)	1.46 [0.85–2.53]	0.168	1.73 [0.92–3.23]	0.124
≥ 3	47 (39.8)	39 (15.9)	4.12 [2.36–7.20]	**<0.0001**	6.03 [3.12–11.65]	**<0.0001**
Gestational age						
1st trimester	20 (16.9)	60 (24.3)	1.00		1.00	—
2nd trimester	44 (37.2)	113 (45.9)	1.17 [0.63–2.16]	0.620	1.22 [0.61–2.48]	0.571
3rd trimester	54 (45.7)	73 (29.6)	2.22 [1.20–4.11]	0.011	2.63 [1.29–5.36]	**0.008**
Previous ANC follow up						
0 ANC	19 (16.1)	14 (5.69)	1.00		1.00	—
1–3 ANC	32 (27.1)	87 (35.3)	0.27 [0.12–0.60]	**0.001**	0.22 [0.09–0.57]	**0.002**
4 ANC	67 (56.7)	145 (58.9)	0.34 [0.16–0.72]	**0.005**	0.32 [0.13–0.77]	**0.011**

COR: crude odds ratio; AAR: adjusted odds ratio; CI: confidence interval. Significant *p* values (*p* < 0.05) are in bold.

## Discussion

This study was conducted to assess the practice of conventional medicine self-medication and associated factors regarding pregnant women attending ANC at Moroccan health centers. As a matter of fact, more than half participants had a full ANC follow-up in their previous pregnancy. The main constraints for avoiding ANC follow-up for the total group were lack of financial and logistical resources. However, women who had at least one ANC were more likely to self-medicate than others. This result could be due to lack of awareness among women about the danger of drugs during pregnancy. ANC plays a crucial role in raising women’s awareness of the different reactions of their new and changing body to drugs and to the transplacental passage of certain molecules (Biblot, 2013).

The present study showed that the prevalence of conventional medicine self-medication during pregnancy was 32%. This finding was in line with the studies carried out in Jordan (33.1%) (Alsous et al., 2021), Serbia (34.7%) (Odalovic et al., 2012), and Pakistan (37.9%) (Bohio et al., 2016). The prevalence was higher than in Indonesia (11.7%) (Atmadani et al., 2020), Netherlands (12.5%) (De Waard et al., 2019), and Egypt (16.6%) (Tawfik et al., 2021) but lower than in Ethiopia (42.2%) (Niriayo et al., 2021), Tanzania (46.2%) (Marwa et al., 2018), and Nigeria (62.9%) (Joseph et al., 2017). This was due to poor awareness in pregnant women regarding medicines (83%) (Alani et al., 2020), poor knowledge about medication use during pregnancy (Alani et al., 2020), and availability of over-the-counter drugs in pharmacies. As in most African countries, health care providers may not adequately educate pregnant women about the potential health risks of self-medication for women and their fetuses due to high workloads.

The principal reason for practicing modern drug self-medication was time-saving. This finding was in line with the study conducted in Ethiopia (73.6%) (Zewdie et al., 2018), where,as in northern Jordan, self-medication was related to previous experience with the disease (47.7%) (Alsous et al., 2021). Moreover, in southern Italy and Egypt, it was related to an assumption that the disease was minor (47%) (Navaro et al., 2018) and (47.8%) (Khalaf et al., 2018). However, in Iran, the main reason was a lack of knowledge about the disease (Baghianimoghadam et al., 2013). Access to medicines without a medical prescription facilitates self-medication.

Modern drug self-medication is practiced mainly in the first quarter, up to 68.6%. This result was different from the studies carried out in Tanzania (Marwa et al., 2018) and France (Cabut, 2017), where self-medication was more frequent in the second and third trimesters, respectively. Self-medication is a consequence of the apparition of physiological symptoms experienced by pregnant women in the first trimester, namely, nausea, vomiting, and headaches. These symptoms diminish during the second trimester and resume in the third trimester. They are combined with other symptoms such as ligament and muscle pain and gastro-esophageal reflux (Elaine et al., 2021). This highlights an alarming threat because exposure to drugs during this period is likely to cause congenital malformations (Bérard et al., 2007; Gidai et al., 2008).

In this study, pregnant women used drugs without any medical advice to treat mainly nausea and vomiting (70.3%), whereas headaches were the main ailment treated in North East Ethiopia (30%) (Tuha et al., 2020), Jordan (85.5%) (Alsous et al., 2021), Europe, North America and South America, and Australia (Lupattelli et al., 2014). Pregnancy is a normal physiological state that leads to pathological changes that must be managed properly, and pregnant women should only take medication by prescription.

Paracetamol was mostly self-used in pregnancy. This result was comparable to the study conducted in Harar town (Jambo et al., 2018). According to the FDA, acetaminophen may be linked to attention deficit hyperactivity disorder (ADHD) in children born to women who take the drug at any time during pregnancy (FDA, 2019). Other studies have shown that it can be associated with neuropsychiatric risks, neurodevelopmental delays (Brandlistuen et al., 2013), autism spectrum disorders (Liew et al., 2016), and even attention-deficit/hyperactivity disorder (Hoover et al., 2015). Therefore, the use of paracetamol during pregnancy should be carefully considered, and pregnant women should always consult their doctors before use.

The use of aspirin during pregnancy is alarming, even though it is a drug that was once classified as probably safe (category D) (Black and Hill, 2003). A study conducted among Swedish pregnant women showed that aspirin users had a higher incidence of intrapartum bleeding [AOR = 1.63; 95% CI (1.30–2.05)], postpartum hemorrhage [AOR = 1.23; 95% CI (1.08–1.39)], and postpartum hematoma [AOR = 2.21; 95% CI (1.13–4.34)], and the risk of a neonatal intracranial hemorrhage was also increased [AOR = 9.66; 95% CI (1.88–49.48)] (Hastie et al., 2021).

These data show that it is mandatory to reinforce the awareness among pregnant women on the risks related to the use of paracetamol and aspirin, especially since they are sold over the counter (Bremer et al., 2017).

The first source of information for conventional medicine self-medication was pharmacist. A similar source of information was reported in several studies conducted in Ethiopia (Sema et al., 2020; Tuha et al., 2020; Niriayo et al., 2021). The pharmacist is often in direct contact with the women when they purchase medicines and should take this opportunity to counsel them about medication use and safety (Michael et al., 2019).

This study highlights age, parity, gestational age, previous ANC follow-up, chronic illness, and drug treatment as the main factors associated with self-medication. More than half of women over 30 years old were more likely to self-medicate than others. This finding is comparable to the study conducted by Atmadani et al. in 2020, which indicated that women over 28 years of age were significantly more likely to self-medicate [AOR = 2.14, 95% CI (1.01–4.50)] (Atmadani et al., 2020). Similarly, multiparity was positively associated with self-medication. This finding agrees with a study conducted in Brazil in 2015 [AOR = 1.25, 95% CI (1.03–1.5)] (Bertoldi et al., 2014). This finding could be due to the experience acquired by these women through drug use, age, and number of pregnancies (Lutz et al., 2020).

Pregnant women with chronic illness and those who were following therapeutic treatments were less likely to administer self-medication compared to their counterparts. This was similar to a finding study conducted in China (Lei et al., 2018).

Although the level of education and occupation of pregnant women were not associated with self-medication, several studies have shown that a low level of knowledge increases the practice of self-medication (Ebrahimi et al., 2017).

This study addressed a knowledge gap regarding self-medication during pregnancy in the Settat region. The results of this study call for spreading more awareness among pregnant women by health care providers and the implementation of adequate strategies to control the marketing, distribution, and use of conventional drugs.

### Study limitations

This study had some limitations, such as the sample size. The sample size needs to be increased in order to reach all social segments of the population. Also, the study can be influenced by social desirability bias and recall bias, which can lead to an underestimation of drug use among women and confusion about the names of drugs. Another limitation was that the survey did not reach pregnant women in the rural area since it was conducted at Settat health centers. This did not allow drawing a conclusion on the behavior of pregnant women toward self-medication.

## Conclusion

Self-medication is a threat to the safety of the developing fetus and the pregnant woman. The prevalence of modern drug self-medication among pregnant women in Morocco is classified as lower than in several African countries such as Egypt and Ghana (Zeid et al., 2020; Gbagbo and Nikrumah., 2020). The associated factors of modern drug self-medication were age, parity, chronic illness, and therapeutic treatment.

The risk of adverse effects or intoxication related to excessive or inappropriate self-medication is well-documented. In view of these data, in the context of the modalities of delivery of medicines in Morocco, it is imperative that information concerning medicines be delivered to pregnant women by the different health professionals involved in the monitoring of pregnant women.

## Data Availability

The original contributions presented in the study are included in the article/Supplementary Material; further inquiries can be directed to the corresponding author.
